# Multiple Sclerosis: Diagnosis, Management, and Future Opportunities

**DOI:** 10.3390/jcm12144558

**Published:** 2023-07-08

**Authors:** Elisabetta Maida, Luigi Lavorgna

**Affiliations:** Department of Advanced Medical and Surgical Sciences, University of Campania Luigi Vanvitelli, 80131 Napoli, Italy; maidaelisabetta92@gmail.com

Multiple sclerosis (MS) is one of the most common inflammatory neurological diseases which leads to a highly heterogeneous set of symptoms and signs due to the differential involvement of the motor, sensory, visual, and autonomic systems [1].

In light of the high incidence and prevalence of the disease, research has always been particularly focused within the field of MS, which has been reflected in a constantly active and buzzing scientific community. In particular, over the past few years several “hot topics” have emerged which are now dominating the international field of research: from the search for biomarkers that could predict the progression of MS, to possible contributors to its pathogenesis, and to the clinical–laboratory management of the patient in order to improve his/her quality of life.

This Special Issue published in the Journal of Clinical Medicine (JCM) is dedicated to addressing the wide-ranging management of MS, covering the most up-to-date topics. I had the pleasure of gathering innovative and high-quality scientific contributions on very diverse topics but all in their own way essential to the advancement of research. Hence, the purpose of this editorial is to summarize the results of the studies presented in this Special Issue.

Within the context of the doctor–patient relationship, Davidescu and colleagues [2] sought to determine whether patients with relapsing–remitting MS (RRMS) presented with more prevalent personality traits compared with a control population. The study’s rationale was that distinct personality traits might be associated with a different means of coping with the disease and its symptoms, and accordingly, the recognition of these profiles can influence the neurologist’s decisions and the patient’s compliance. A total of 122 patients were randomized into two groups and underwent the DECAS Personality Inventory test. The results showed a lower prevalence of the trait of “extroversion” in subjects with MS than in those in the control group, particularly in individuals with greater disability (as measured by the Expanded Disability Status Scale (EDSS)). Higher EDSS also correlated with both avoidant and melancholic personality traits; moreover, in relation to proactivity, MS patients were found to be more passive but also more compassionate than others. The topic of patients’ emotional response was further addressed by Rosa et al. [3] who performed a longitudinal study on changes in the health-related quality of life (HRQoL) of patients with early-onset MS between the ages of 12 and 25 years. Health-related quality of life in the domain of physical activity has been found to be a predictor of disability progression in people with RRMS [4]. At baseline and at the end of the fourth year, patients were asked to complete the Pediatric Quality of Life inventory (PedsQL) test, which consists of 23 questions divided into 4 subscales: physical functioning, social functioning, emotional functioning, and finally work/school functioning. Notably, the results showed a decrease in the emotional functioning subscale and concomitantly an increase in the social functioning subscale, which also appeared to be affected by the occurrence of relapse.

The implementation of MS patient management and doctor–patient relationship includes Digital Health; telemedicine, has indeed proven to be effective in the management of many chronic diseases [5], including through wearable devices or commonly used applications such as Google Maps [6,7]. The study by Jerkovic et al. [8] investigated the introduction of an electronic self-assessment scale through which patients with MS can assess their disability and convey their symptoms to the neurologist, the electronic Patient-Reported Expanded Disability Status Scale (ePR-EDSS). ePR-EDSS is highly correlated with EDSS and may enable valuable clinical information to be detected remotely even before the in-person visit. The questionnaire revealed greater issues in patients with progressive forms of MS, compared with those with RRMS regarding both overall well-being, strength, coordination, the genitourinary system and fatigue.

Since there is no definitive treatment for MS, countless studies have focused on finding the optimal treatment strategy. The efficacy of Natalizumab (NTZ) and Fingolimod (FTY) has been amply demonstrated [9,10]; however, the possible occurrence of progressive multifocal leukoencephalopathy, the risk of lymphopenia-related infections and cardiologic adverse events are reasons for the discontinuation of these treatments [11]. Rituximab (RTX) has also been found effective in controlling inflammatory disease activity as well as the occurrence of new lesions [12]. The study by Rocio Hernández-Preciado et al. [13] compared the effect of RTX with NTZ and FTY in a cohort of subjects with RRMS. Although there was some discordance among the cohorts, RTX proved to be more effective in terms of relapse control; moreover, there was a reduction in the average EDSS score against the increase reported in the group treated with NTZ or FTY. Among the available treatments for MS to date, Ocrelizumab (OCR) certainly has a leading role, as it is approved for both RRMS and the secondarily and primarily progressive forms (SPMS and PPMS) [14,15,16]. The aim of the work of Lanzillo and colleagues [17] was to collect real-word data on OCR and to explore possible prognostic predictors of clinical outcome. For this purpose, data were collected from 383 patients with RRMS, SPMS and PPMS, one year before the start of OCR (T-1), at the beginning of therapy (T0) and one year later (T1). The patients with a disease duration of less than 10 years showed an increase in EDSS from T-1 to T0 without a further increase between T0 and T1, in contrast to those with a disease duration of more than 10 years, who also displayed an increase in the T0–T1 period. Similarly, patients with an EDSS less than 4 showed no variation in the score, while patients with an EDSS greater than 4 showed an increase from T-1 to T0. Finally, patients with RRMS showed no change in the EDSS between T-1 and T0 nor between T0 and T1, whereas patients with SPMS and PPMS exhibited an increase in the EDSS from T-1 to T0. These results seem to support early treatment with OCR, from the earliest stages of the disease.

A relevant issue to consider before starting a treatment with an immunosuppressant is the possible occurrence and impact of side effects. Among these side effects it is essential JC Polyomavirus (JCPyV) positivity due to the possibility of developing progressive multifocal leukoencephalopathy (PML), which can be a major risk to the lives of these patients. The study conducted by Prezioso and his group [18] evaluated different types of JCPyV markers such as JCPyV DNA and microRNA, anti-JCV index and noncoding control region (NCCR) sequence in urine and plasma. These parameters were evaluated before the start of treatment with NTZ, OCR, dimethylfumarate and FTY, after 6 months (T6) and at 12 months (T12) and subsequentially compared with a control group of healthy subjects. The results showed increased viremia and urinary excretion of JCPyV DNA at T6 and T12 in subjects with MS, but not in healthy controls. The NCCR archetype was detected in all positive urine samples, while mutations resulting in a more neurotrophic variant were observed in plasma. Additionally, the copy number of microRNA in the urine and plasma of MS patients were found to be increased after treatment, while they remained unchanged in the control group samples. The conclusion is that analysis of JCPyV DNA copies and microRNA levels may allow for the monitoring of JCPyV activity, more effectively even than the anti-JCV index, and may predict which patients with MS are at higher risk of developing PML. Sticking to the area of adverse reactions, Sirbu and colleagues [19] presented the cases of two patients treated with OCR and NTZ who developed dermatological issues. Specifically, skin biopsy results showed that the first patient suffered from nummular, diffuse eczema on the trunk and limbs, while the patient treated with NTZ experienced chronic spongiotic dermatitis. The report highlights how it is of utmost importance to tailor treatment according to the individual’s characteristics, comorbidities and medical history to maximize patient compliance and minimize the risks. Lastly, the authors of this case series report [20] brought their experience regarding disease reactivation in patients with SPMS treated with Siponimod (SIP). SIP is a relatively new drug that has become quickly popularized in the MS market, posing as an alternative to its big brother, FTY. For this reason, long-term data are not yet available. This report highlighted the possibility of disease reactivation after switching from FTY to SIP. However, further studies are needed to investigate the safety and efficacy of this therapeutic switch and to better understand the molecular mechanism underlying MS reactivation.

In the management of the MS patient, it is of great importance to specifically address motor disabilities as they have a high impact on the quality of life. The study by Molina-Rueda et al. [21] sought to analyze and evaluate spatiotemporal, kinetic and kinematic gait parameters of patients with MS in order to better understand their motor disabilities to be able to provide a highly personalized rehabilitation program. A control group of 10 healthy subjects and 8 subjects with RRMS with mild disability were compared. The results indicated that subjects with MS with mild disability walked with similar joint angles and moments than controls; moreover, no changes in spatiotemporal parameters were recorded. Regarding kinetic parameters, subjects with MS with mild disability showed changes in timing in the most affected lower limb. Thus, the study supports the employment of rehabilitation techniques from the earliest stages of the disease, as changes in gait parameters are already detected. Finally, another study [22] evaluated the effect of Reflex Locomotion Therapy (RLT) on balance, gait and fatigue in MS patients. RLT is a rehabilitation method that relies on activating the preorganized circuits of the central nervous system (CNS) through specific positions and pressing on specific points in certain directions, thus triggering motor programs with locomotion components. The findings revealed a significant improvement in balance, measured by the Berg Balance Scale and the balance subsection of the Performance-Oriented Mobility Assessment (POMA), while also showing an increase in gait performance, measured by the gait subsection of POMA. An analysis of spatiotemporal parameters demonstrated a significant improvement in stride length and speed, as well as time with double stance. Finally, concerning the kinematic parameters, the analysis showed a significant change in hip and knee joint movements.

Last but not least, two papers [23,24] in this Special Issue focused on molecular research to better understand the pathogenetic mechanisms of MS. The study designed by Barac and colleagues [23] aimed to assess the potential contribution in MS susceptibility of polymorphisms of IL-27 and IL-23 genes, both modulators of T-helper 17 (Th17) cell activity. A total of 157 people diagnosed with MS and 95 healthy controls were enrolled, and their genotypes for IL-27 T4730C, IL-27 A964G and IL-23 receptor gene G1142A were ascertained. The IL27-T4730C gene polymorphism was found to be significantly associated with increased odds of MS with a dominant genetic pattern. Meanwhile, subjects carrying the IL-27 A924G (AG + GG) variant had a higher likelihood of MS than noncarriers. In contrast, SNPIL-23-R381Q conferred a lower likelihood of MS according to a codominant model of inheritance and the allelic model. Zarobkiewicz et al. [24] investigated the role of the platelet endothelial cell-adhesion molecule (PECAM-1/CD31), a protein involved in several mechanisms such as leukocyte transmigration, apoptosis, angiogenesis and many others. In MS patients, the expression of PECAM-1 is up-regulated in serum, plasma and cerebrospinal fluid and may also be involved in stabilizing the blood–brain barrier. γδ T cells, particularly the Vδ1 subgroup, also express PECAM-1 protein. Notably, based on flow cytometry data, PECAM1 was modulated differently on γδ T cells: it was up-regulated during relapse, but down-regulated during clinical remission phases. In addition, significant down-regulation of CD3 expression was observed on γδ T cells of MS patients, particularly during relapse. This might suggest an overall activation of γδ T cells during the course of multiple sclerosis.

In conclusion, this Special Issue has collected several interesting papers that will help neurologists in the process of MS patient management and treatment choices. I am grateful to the JCM team for their continuous support of this Special Issue and to the reviewers for their highly professional feedback. Lastly, I would like to extend my thanks to all the authors for their valuable contributions.

## Data Availability

Data sharing not applicable.
